# Empirical therapy for hepatic lesions in suspected clonorchiasis: a comparative efficacy analysis of Albendazole and Praziquantel

**DOI:** 10.3389/fphar.2026.1878617

**Published:** 2026-07-08

**Authors:** Ya-zhao Cao, Peng-zhen Guo, Yu-feng Zhang, Wen-yu Li, Qi-fa Shen, Zhi-hui Li, Zi-ying Lei, Jun-feng Chen, Jing Zhang

**Affiliations:** 1 Department of Infectious Diseases, The Third Affiliated Hospital of Sun Yat-sen University, Guangzhou, Guangdong, China; 2 Department of Internal Medicine II, Guangzhou Guanggang New Town Hospital, Guangzhou, Guangdong, China; 3 Department of Infectious Diseases, Guangzhou Panyu Maternal and Child Health Care Hospital, Guangzhou, Guangdong, China

**Keywords:** albendazole, clonorchiasis, diagnostic certainty, hepatic space-occupying lesions, praziquantel, retrospective cohort study

## Abstract

**Background:**

Clonorchiasis frequently presents as intrahepatic space-occupying lesions, posing a diagnostic challenge due to the low sensitivity of fecal examination. While Praziquantel is the standard first-line therapy, Albendazole is often used empirically in suspected cases. However, the comparative efficacy of these two agents across different levels of diagnostic certainty (confirmed vs. suspected) remains poorly defined.

**Methods:**

This retrospective cohort study included 151 patients with clonorchiasis-related hepatic lesions treated at our institution between January 2018 and December 2025. Patients were categorized into a Praziquantel group (*n =* 78) and an Albendazole group (*n =* 73). The primary outcome was clinical cure within 90 days, defined as symptom resolution, lesion reduction ≥50%, and normalization of eosinophil counts. Multivariable logistic regression and Generalized Estimating Equations (GEE) were employed to evaluate the interaction between treatment regimens and diagnostic certainty.

**Results:**

The overall clinical cure rate was higher in the Praziquantel group (76.9%, 60/78) than in the Albendazole group (67.1%, 49/73), though the difference was not statistically significant (*χ* ^2^ = 1.804, *P* = 0.179). Stratified analysis revealed that Praziquantel was significantly more effective in confirmed cases (90.0% vs. 62.5%, *P* = 0.047), whereas no significant difference was observed in suspected cases (68.8% vs. 68.4%, *P* = 0.971). The “Drug/times Diagnosis” interaction test showed marginal significance (*P =* 0.064). After adjusting for confounders, Albendazole showed similar clinical outcomes to Praziquantel (Adjusted OR = 0.665, 95% CI: 0.298–1.482, *P =* 0.318). GEE analysis confirmed a significant “Drug × Time” interaction (*P <* 0.001), indicating distinct kinetic patterns of recovery, with Praziquantel demonstrating a superior cure advantage over time. Age was identified as an independent predictor of clinical cure (*P =* 0.017).

**Conclusion:**

The efficacy of Albendazole in treating clonorchiasis-related hepatic lesions is modulated by diagnostic certainty. While Praziquantel remains the superior choice for confirmed cases, Albendazole demonstrates comparable clinical efficacy in stool-negative suspected cases. These findings indicate that Albendazole demonstrates similar lesion resolution to Praziquantel in stool-negative suspected cases. However, given the retrospective design, these results should be interpreted as hypothesis-generating, necessitating prospective trials for confirmation.

## Introduction

Clonorchiasis, caused by the trematode *Clonorchis sinensis*, is a major foodborne parasitic disease and has been classified as a Group 1 carcinogen by the International Agency for Research on Cancer (IARC) (Qian et al., 2016). Despite global control efforts, it remains a significant public health challenge in East Asia, particularly in Guangdong Province, China (Chai and Jung, 2019). The Pearl River Delta (PRD) region maintains a high prevalence of infection due to the deeply entrenched cultural practice of consuming raw freshwater fish (Huang et al., 2017; Zhang et al., 2024). Pathologically, chronic infection triggers periductal fibrosis, biliary hyperplasia, and focal eosinophilic infiltration (FEI) (Liao et al., 2006). These changes often manifest radiographically as intrahepatic space-occupying lesions, which can closely mimic the presentation of cholangiocarcinoma or metastatic tumors, leading to diagnostic confusion (Lan et al., 2023).

The clinical diagnosis of clonorchiasis is significantly hindered by the low sensitivity of stool microscopy—the conventional diagnostic method—especially in patients with light parasitic loads or those suffering from biliary obstruction (Keiser et al., 2010). Consequently, false-negative results are frequent, leaving a substantial proportion of symptomatic patients without a parasitological diagnosis (Qian et al., 2024).

Praziquantel is the established first-line treatment, demonstrating high efficacy against adult flukes; however, its effectiveness against juvenile stages is notably limited (Lee et al., 2024). In real-world clinical settings, when faced with patients exhibiting hepatic lesions, peripheral eosinophilia, and a clear history of raw fish consumption—yet negative stool tests—clinicians often resort to empirical therapy. In these scenarios, Albendazole, a broad-spectrum benzimidazole, is frequently utilized off-label (Qian and Zhou, 2021). Despite its common use, direct comparative evidence evaluating the efficacy of these two drugs remains scarce, particularly regarding how diagnostic certainty (confirmed vs. suspected cases) and the specific pathology of hepatic lesions influence treatment outcomes.

Therefore, this study aims to evaluate the comparative clinical efficacy of Praziquantel versus Albendazole in patients presenting with *C. sinensis*-related hepatic lesions. We hypothesize that the therapeutic response is modulated by the level of diagnostic certainty and that the two regimens exhibit divergent longitudinal patterns in the resolution of lesions over time.

## Methods

### Study design and population

This single-center retrospective cohort study was conducted using data retrieved from the electronic medical record system of the Third Affiliated Hospital of Sun Yat-sen University. The study protocol received ethical approval from the Institutional Review Board (IRB) of the hospital (Approval No. ZSSFYY-2026–031-01). The requirement for informed consent was waived due to the retrospective nature of the study and the fact that it did not interfere with routine clinical management.

Adult patients admitted to the Department of Infectious Diseases or the Department of Hepatology at our institution between January 2018 and December 2025 were screened. The inclusion criteria were: (1) age > 18 years; (2) presence of one or more intrahepatic space-occupying lesions confirmed by computed tomography (CT) or magnetic resonance imaging (MRI); (3) absolute peripheral blood eosinophil count > 0.5 × 10^9^/L (Chen et al., 2018); (4) a clear history of raw freshwater fish consumption and/or long-term residence in endemic regions (e.g., Guangdong or Guangxi provinces) (Li X. et al., 2025); (5) administration of standardized monotherapy with either Praziquantel or Albendazole; and (6) possessed complete baseline information along with imaging and laboratory follow-up data within 3 months after treatment. Patients were excluded if they met any of the following criteria: (1) confirmed co-infection with other parasites (e.g., Paragonimus or Schistosoma); (2) concomitant malignancy, autoimmune liver disease, or drug-induced liver injury; (3) combined use of other anthelmintics or corticosteroids during the treatment period; or (4) absence of critical follow-up data, precluding the assessment of clinical outcomes.

Based on parasitological evidence, patients were stratified into two diagnostic tiers: Definite Group: Defined by at least one positive stool examination for *C. sinensis* eggs. Suspected (Presumptive) Group: Defined by three consecutive negative stool examinations despite exhibiting typical clinical and radiological features—including persistent eosinophilia, periductal enhancing lesions on imaging, and a documented history of epidemiological exposure—with no other identifiable etiology.

### Exposure and treatment regimens

The primary exposure of interest was the type of anthelmintic agent administered: Praziquantel or Albendazole. The Praziquantel regimen consisted of 20 mg/kg per dose, three times daily for 3 consecutive days (Qian et al., 2013a). The Albendazole regimen was administered at a dosage of 20 mg/kg per day in 2–3 divided oral doses for 7 days. A small subset of patients received an extended course of up to 14 days; these cases were included in the Albendazole group due to the limited sample size (Qian et al., 2024; Xiao et al., 2011; Qian et al., 2022). Furthermore, we conducted a sensitivity analysis excluding the 14-day subgroup, and the results remained consistent (data not shown). All subjects received monotherapy without the co-administration of other antiparasitic agents.

### Outcome definitions

The primary outcome was clinical cure within 90 days post-treatment, defined by the simultaneous fulfillment of the following three criteria: (1) Symptomatic relief: Complete resolution or significant improvement of baseline gastrointestinal symptoms (e.g., right upper quadrant pain, abdominal distension, and dyspepsia). (2) Radiological response: Follow-up imaging (liver ultrasound, CT, or MRI) demonstrating a ≥50% reduction in the sum of the diameters of target lesions—classified as Partial Response (PR)—or their complete disappearance—classified as Complete Response (CR). (3) Laboratory normalization: Normalization of the peripheral blood eosinophil count (< 0.5 × 10^9^/L).

Imaging assessments were performed independently by two radiologists who were blinded to the treatment assignments. Any discrepancies were resolved through consensus or by adjudication from a third senior radiologist. Laboratory parameters were obtained from the official reports issued by the hospital’s clinical laboratory.

### Statistical analysis

Continuous variables were expressed as mean ± standard deviation or median (interquartile range, IQR), with normality assessed via the Shapiro–Wilk test. Comparisons between groups were performed using the independent samples t-test or Mann–Whitney U test, as appropriate. Categorical variables were presented as frequencies (percentages) and analyzed using the Chi-square test or Fisher’s exact test. Clopper–Pearson intervals were calculated for initial categorical analysis, while Wilson score intervals were used for longitudinal cure rate plots to ensure binomial proportion accuracy. To evaluate effect modification, a multivariable logistic regression model incorporating a multiplicative interaction term (Treatment × Diagnostic Certainty) was fitted. The significance of the interaction was assessed using the Wald test, with *P <* 0.05 indicating significant heterogeneity in treatment effects across diagnostic subgroups. Multivariable logistic regression was further employed to estimate the association between treatment and clinical cure, calculating adjusted odds ratios (aORs) and 95% confidence intervals (CIs). Predefined covariates included age, sex, diagnostic category (definite vs. suspected), baseline white blood cell count, eosinophil count, alanine aminotransferase (ALT), number of lesions, and maximum lesion diameter. For the repeated measurements at 30, 60, and 90 days, Generalized Estimating Equations (GEE) with a logit link function and binomial distribution were utilized to account for within-subject correlation. An exchangeable correlation structure was specified. The model was adjusted for age, sex, baseline laboratory indicators (ALT, EOS, WBC), and lesion characteristics. All tests were two-sided, and *P <* 0.05 was considered statistically significant. Data analysis was performed using the SPSSpro platform (https://www.spsspro.com/).

## Results

### Baseline characteristics and histopathological findings

A total of 151 patients were enrolled, including 78 in the Praziquantel group and 73 in the Albendazole group. The two groups were comparable in terms of age, sex, pre-treatment white blood cell count, eosinophil count, lesion number and size, ALT, and AST levels (all *P >* 0.05). However, a significantly higher proportion of definite cases was observed in the Praziquantel group (38.5%) compared to the Albendazole group (21.9%) *(P =* 0.027), which was identified as a potential confounding factor (Table 1).

**TABLE 1:** Baseline characteristics of the study population (*N =* 151).

Variable	Total (*N =* 151)	Praziquantel	Albendazole	P value
		(*n =* 78)	(*n =* 73)	
Age, median (Q1-Q3) (years)	34 (28–42.5)	33 (27.25–41.75)	34 (29–43)	0.504
Male sex, n (%)	128 (84.8%)	66 (84.6%)	62 (84.9%)	0.957
Definite diagnosis^a^, n (%)	46 (30.5%)	30 (38.5%)	16 (21.9%)	0.027*
WBC,median (Q1-Q3) × 10^9^/L	15.31 (10.90–20.98)	14.56 (11.35–20.11)	16.25 (10.37–21.27)	0.76
Eosinophil count, median (Q1-Q3) × 10^9^/L	7.26 (3.75–13.53)	7.25 (3.83–12.91)	8.64 (3.68–14.66)	0.776
Number of liver lesions, median (Q1-Q3)	3 (2–3)	3 (2–3.75)	3 (1–3)	0.3
Lesion size^b^, median (Q1-Q3) cm	29 (22–37)	27 (23–36)	29 (22–41)	0.367
ALT, median (Q1-Q3) U/L	68 (42–99.5)	70 (43.5–109)	63 (38–92)	0.282
AST, median (Q1-Q3) U/L	35 (26–50)	35 (26.25–45.5)	37 (26–54.3)	0.73
GGT, median (Q1-Q3) U/L	119 (67.5–178.5)	127.5 (84–186.75)	113 (63–159)	0.141
ALP, median (Q1-Q3) U/L	163 (115–232)	171.5 (115.25–245)	161 (116–203)	0.387

^a^ Definite diagnosis: positive stool examination for *Clonorchis sinensis* eggs.

^b^ Lesion size: Calculated based on the maximum diameter of the largest lesion.

^c^ Statistical analysis: Continuous variables compared by t-test (normal) or Mann–Whitney U test (non-normal); categorical variables by *χ*2 test.

* *P* < 0.05.

**FIGURE 1:**
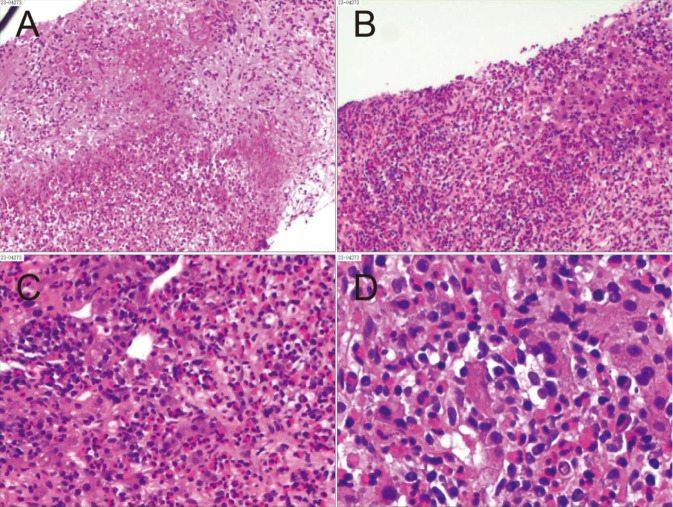
Histopathological features of liver tissue with H&E staining. **(A)** Focal necrotic lesion characterized by dense inflammatory infiltration and disruption of the hepatic lobular architecture (×100). **(B)** Peripheral zone of the lesion showing degenerated and necrotic hepatocytes (×100). **(C)** Diffuse inflammatory cell infiltration predominantly composed of eosinophils (×200). **(D)** High-magnification view showing typical morphological features of eosinophils; no parasite components or Charcot-Leyden crystals were identified (×400).

Hepatic ultrasound revealed that the lesions were frequently multiple hypoechoic nodules with ill-defined margins, predominantly distributed in the peripheral liver. These imaging characteristics closely mirrored the pathological findings of marked eosinophilic infiltration observed in representative cases. Following anthelmintic therapy, a dynamic evolution was observed: lesions either reduced significantly in size (e.g., from 21 × 17 mm to 4 × 3 mm) or demonstrated complete absorption (e.g., a 20 × 17 mm lesion resolved entirely). Histopathological examination (HE staining) of liver tissue sections revealed focal necrotic lesions with disrupted lobular architecture and marked inflammatory infiltration (Figure 1). The infiltrates consisted predominantly of eosinophils, accompanied by scattered lymphocytes and plasma cells. Hepatocellular degeneration and necrosis were evident within the lesion areas. No parasite ova, worms, or Charcot-Leyden crystals were observed, and no obvious granuloma formation or portal fibrosis was detected.

### Overall clinical cure rates showed no significant difference

**FIGURE 2:**
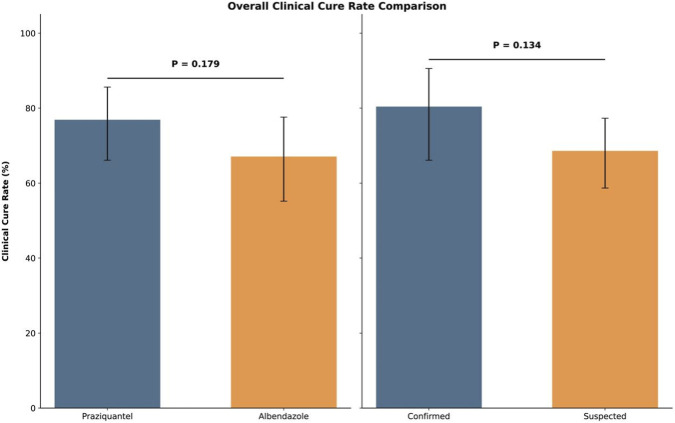
Comparison of clinical cure rates across treatment groups and diagnostic categories. Clinical cure rates are presented as percentages, with error bars representing the 95% confidence intervals (CIs). The overall cure rate was higher in the Praziquantel group (76.9%) than in the Albendazole group (67.1%), though the difference was not statistically significant (*P =* 0.179). Stratified analysis demonstrated no significant difference in cure rates between confirmed (80.4%) and suspected (68.6%) cases (*P =* 0.134).

In the overall efficacy comparison, the clinical cure rate in the Praziquantel group (76.9%, 60/78; 95% CI: 66.1%–85.6%) was higher than that in the Albendazole group (67.1%, 49/73; 95% CI: 55.2%–77.6%), though the difference was not statistically significant (*χ* ^2^ = 1.804, *P =* 0.179). Additionally, no significant difference was observed between the overall cure rates of the definite group (80.4%, 37/46; 95% CI: 66.1%–90.6%) and the suspected group (68.6%, 72/105; 95% CI: 58.7%–77.3%) (*χ* ^2^ = 2.242, *P =* 0.134) (Figure 2).

### Therapeutic potential of albendazole in suspected cases

**FIGURE 3:**
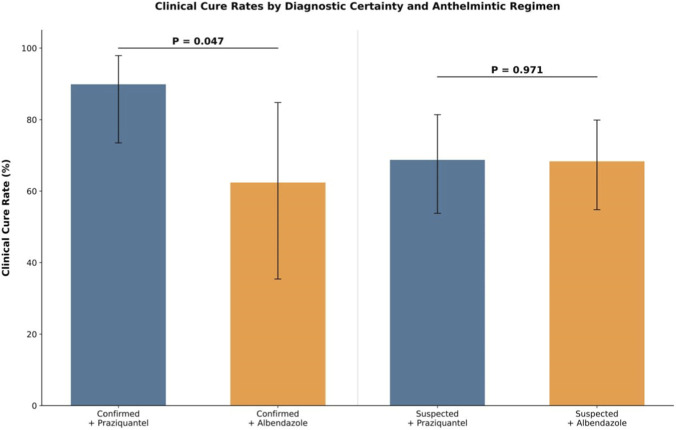
Clinical cure rates stratified by diagnostic certainty and anthelmintic regimen. Data are presented as clinical cure rates (%) with 95% confidence intervals (CIs) represented by error bars. Among confirmed cases, Praziquantel demonstrated a significantly higher cure rate compared to Albendazole (90.0% vs. 62.5%; *P =* 0.047). In contrast, no significant difference was observed between the Praziquantel and Albendazole groups in suspected cases (68.8% vs. 68.4%; *P =* 0.971).

Stratified analysis by diagnostic certainty revealed that in the definite subgroup, the clinical cure rate was significantly higher in the Praziquantel group (90.0%, 27/30; 95% CI: 73.5%–97.9%) than in the Albendazole group (62.5%, 10/16; 95% CI: 35.4%–84.8%) (*P =* 0.047, Fisher’s exact test). Notably, the confidence interval for Albendazole in this subgroup was wide, reflecting the limited sample size. Conversely, in the suspected subgroup, cure rates were nearly identical between the Praziquantel (68.8%, 33/48; 95% CI: 53.8%–81.4%) and Albendazole (68.4%, 39/57; 95% CI: 54.8%–79.9%) groups (*χ* ^2^ = 0.001, *P =* 0.971) (Figure 3).

An interaction term (Treatment × Diagnostic Certainty) was included in the multivariable logistic regression model to assess effect modification. The results indicated a marginally significant trend (*P =* 0.064), suggesting a potential difference in treatment efficacy between definite and suspected cases, although it did not reach the conventional level of statistical significance. The odds ratio (OR) for the interaction term was 5.318 (95% CI: 0.906–31.212) (Table 2).

**TABLE 2:** Assessment of the interaction effect between treatment regimen and diagnostic certainty on clinical cure.

Variable	β (Coefficient)	SE	Wald	P	OR (95% CI)
			*χ* ^2^		
Main effects					
Treatment (praziquantel vs. Albendazole)	0.015	0.422	0.001	0.971	1.015 (0.444–2.322)
Diagnostic certainty (confirmed vs. Suspected)	−0.262	0.59	0.198	0.656	0.769 (0.242–2.444)
Interaction term					
Treatment × diagnostic certainty	1.671	0.903	3.426	0.064	5.318 (0.906–31.212)

This model assesses whether the efficacy of the treatment varies by diagnostic certainty. The interaction term showed a marginal trend towards significance (*P =* 0.064).

### Independent predictors of clinical cure

**FIGURE 4:**
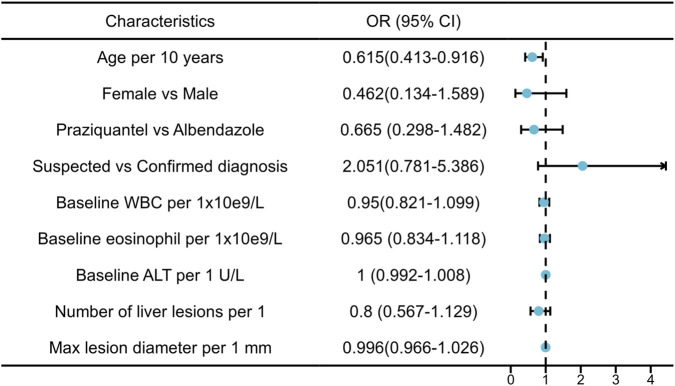
Predictors of clinical cure identified via multivariable analysis. Forest plot displaying adjusted odds ratios (aORs) and 95% confidence intervals (CIs) for factors associated with clinical cure in 151 patients with *C. sinensis*-related hepatic lesions. The vertical dashed line represents the null effect (aOR = 1.0). Age was identified as an independent predictor of treatment outcome (*P =* 0.017).

In the unadjusted univariate analysis, the Albendazole group showed a lower cure rate than the Praziquantel group (Crude OR = 0.613, 95% CI: 0.290–1.298, *P =* 0.179), which was not statistically significant. Multivariable logistic regression revealed that after adjusting for age, sex, diagnostic certainty, ALT, and lesion characteristics, the treatment regimen was not an independent factor influencing clinical cure (Adjusted OR = 0.665, 95% CI: 0.298–1.482, *P =* 0.318). This indicates that Albendazole yields similar clinical responses to Praziquantel after controlling for baseline biases. Notably, age was identified as an independent predictor of cure (Adjusted OR = 0.615, *P =* 0.017), suggesting that clinical cure rates decrease with advancing age (Figure 4).

### Robustness of efficacy confirmed by sensitivity analysis

**FIGURE 5:**
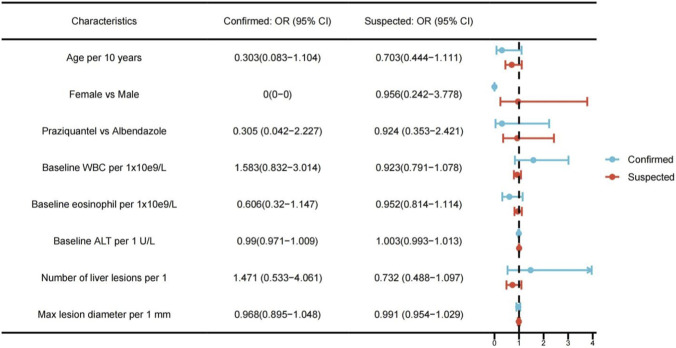
Sensitivity analysis of clinical cure stratified by diagnostic subgroups. Forest plot displaying adjusted odds ratios (aORs) and 95% confidence intervals (CIs) comparing treatment efficacy between confirmed (blue) and suspected (red) cases. The analysis accounts for covariates including demographics, treatment allocation, and baseline clinical characteristics to confirm the robustness of the findings across diagnostic tiers.

To exclude interference from the uneven distribution of diagnostic certainty, a stratified sensitivity analysis was performed. After adjusting for covariates, no significant difference in efficacy was found between Albendazole and Praziquantel in either the definite subgroup (aOR = 0.305, *P =* 0.242) or the suspected subgroup (aOR = 0.924, *P =* 0.872). This further confirms the stability of Albendazole’s efficacy across different diagnostic tiers. (Table 3; Figure 5).

**TABLE 3:** Sensitivity analysis of drug efficacy across different diagnostic subgroups.

Subgroup	Sample size (N)	Adjusted OR	95% CI	P-value
Confirmed cases	46	0.305	0.042–2.227	0.242
Suspected cases	105	0.924	0.353–2.421	0.872

Praziquantel vs. Albendazole.

### Temporal trends in cure rates and time-dependent efficacy

Generalized Estimating Equations (GEE) analysis addressed within-subject correlations and revealed a significant Treatment × Time interaction (*P* < 0.001), indicating that the efficacy trends of the two drugs differed over time. Analysis of main effects showed that the overall cure rate in the Praziquantel group was significantly superior to the Albendazole group (OR = 10.278, 95% CI: 4.123–25.62, *P* < 0.001), suggesting that the curative advantage of Praziquantel is more than 10-fold that of Albendazole after controlling for the time factor (Table 4).

**TABLE 4:** Generalized estimating equation (GEE) analysis of factors associated with clinical cure.

Variable name	Coefficient	Std. Error	P-value	OR (95%CI)
Treatment * Time_Group	−2.055	0.252	0.000***	0.128 (0.078–0.21)
Treatment_Praziquantel	2.33	0.466	0.000***	10.278 (4.123–25.62)
Age per 10 years	0.008	0.131	0.949	1.008 (0.78–1.303)
Group_Suspected	0.014	0.298	0.963	1.014 (0.565–1.819)
Gender_Female	−0.449	0.366	0.22	0.638 (0.311–1.308)
Baseline WBC per 1x10e9/L	0.05	0.053	0.351	1.051 (0.948–1.166)
Baseline eosinophil per 1x10e9/L	0.009	0.057	0.875	1.009 (0.902–1.128)
Baseline ALT per 1 U/L	−0.001	0.002	0.617	0.999 (0.995–1.003)
Number of liver lesions per	0.056	0.111	0.61	1.058 (0.851–1.315)
Max lesion diameter per 1 mm	−0.004	0.009	0.664	0.996 (0.979–1.014)

OR, Odds Ratio. CI, confidence interval.

Reference Groups: Albendazole for Treatment; 30 Days for Time Group.

*** indicates P < 0.001.

**FIGURE 6:**
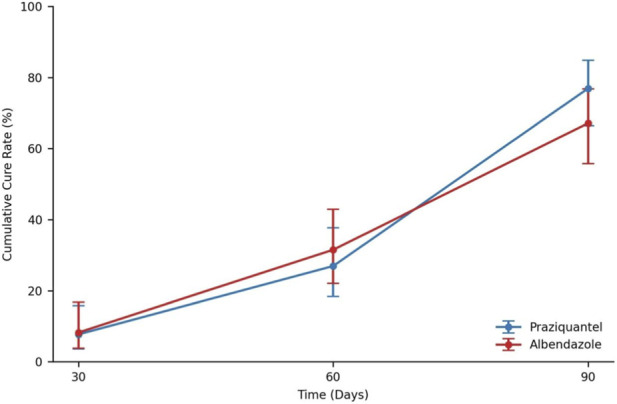
Cumulative clinical cure rates of praziquantel versus albendazole at 30, 60, and 90 days of follow-up. Data are presented as cumulative percentages, with 95% confidence intervals (CIs) calculated using the Wilson score method. The blue line represents the praziquantel group (*n =* 78), and the red line represents the albendazole group (*n =* 73). A progressive upward trend in cure rates is observed in both groups over the 90-day follow-up period.

The cumulative cure rates in both groups showed a progressive upward trend (Figure 6). At 30 days, cure rates were 7.7% (95% CI: 3.0%–16.0%) for Praziquantel and 8.2% (95% CI: 3.2%–17.0%) for Albendazole (*P* > 0.05). By 60 days, rates rose to 26.9% (95% CI: 17.9%–38.0%) and 31.5% (95% CI: 21.5%–43.3%), respectively, with overlapping confidence intervals. At 90 days, the cumulative cure rate was significantly higher in the Praziquantel group (76.9%, 95% CI: 66.2%–85.0%) compared to the Albendazole group (67.1%, 95% CI: 55.4%–77.0%).

### Safety and tolerability profile

Adverse events were rare and mild. Only one patient in the Albendazole group reported a transient elevation of serum creatinine, while no adverse events were observed in the Praziquantel group. No serious adverse events (SAEs) occurred in either group.

## Discussion

This retrospective analysis included 151 patients with hepatic space-occupying lesions associated with Clonorchis sinensis. Our stratified analysis demonstrated that diagnostic certainty greatly affects the interpretation of treatment outcomes. As reported in local epidemiological studies, most participants were adult males, which is closely related to the prevalent habit of consuming raw freshwater fish in endemic areas of Guangdong (Qian et al., 2013b).

Overall cure rates were numerically higher in the praziquantel group. The significant treatment-by-time interaction from GEE analysis revealed a cumulative therapeutic advantage of praziquantel (OR = 10.278), yet this benefit was mainly confined to parasitologically confirmed cases (90.0% vs. 62.5%). For stool-negative suspected cases, the two drugs delivered similar clinical responses (68.8% vs. 68.4%), with no statistically significant difference. This discrepancy across subgroups explains why multivariable logistic regression showed no obvious between-group difference after adjusting for confounders. The strong OR value in the longitudinal GEE model reflects heterogeneous treatment effects rather than universal superiority of praziquantel. Our findings suggest albendazole may have potential value for infections involving larvae or low parasite burdens. Nevertheless, given the retrospective design, small sample sizes in subgroups and wide confidence intervals, these results are purely exploratory and cannot support its use as a standard alternative therapy.

The differing treatment profiles stem from distinct pharmacological mechanisms. Praziquantel effectively eliminates adult liver flukes by disrupting parasite tegument and inducing calcium influx, but it has minimal activity against juvenile worms (Qian et al., 2024). Rapid lysis of adult parasites may also trigger antigen release and aggravate hepatic eosinophilic infiltration (Lee et al., 2024; Ma et al., 2026). In contrast, albendazole and its active metabolite bind to parasite β-tubulin, inhibiting the growth of both adult and larval stages (Qian et al., 2016; Zhang et al., 2019; Xu et al., 2011). Its metabolites may also modulate host inflammatory reactions and relieve eosinophilic infiltration in liver tissue (Qian et al., 2016; Qian et al., 2024). Such dual properties enable albendazole to produce similar therapeutic performance in patients with inflammatory lesions caused by larval migration or mild infection (Li et al., 2019; Li W. et al., 2025). Additionally, its broad-spectrum anthelmintic effect offers advantages for patients with potential mixed fluke infections. Age was also identified as an independent influencing factor; lower cure rates in elderly patients may be attributed to immunosenescence and biliary fibrosis.

This study has multiple inherent limitations that require full consideration. First, treatment regimens were determined by clinicians’ judgment instead of random allocation, leading to inevitable selection bias, even after multivariate adjustment. Second, the suspected case group lacked parasitological evidence and was not a homogeneous population, which may include patients with other eosinophilic liver diseases. Third, liver histopathology only presented non-specific parasitic eosinophilic inflammation; no adult worms, eggs or characteristic periductal fibrosis were found, so histological results alone cannot confirm clonorchiasis. Finally, the retrospective nature caused incomplete documentation of adverse events, which may underestimate the safety profile. Only one transient serum creatinine elevation was observed in the albendazole group, and no serious adverse events occurred in either group.

In clinical practice, praziquantel remains the well-established first-line treatment for parasitologically confirmed clonorchiasis. For patients with typical exposure history, hepatic lesions, eosinophilia but negative stool tests, albendazole can be considered for empirical use. Serological tests also have limitations in distinguishing active and past mild infections (Pan et al., 2024; Li et al., 2018). Previous studies have reported that standard high-dose praziquantel may cause tolerability concerns and potentially compromise medication adherence, while albendazole is associated with lower treatment costs (Zhu et al., 2025; He et al., 2022), an issue that could not be assessed in this retrospective study. In summary, our findings provide exploratory evidence for stratified medication strategies. Large-scale prospective randomized controlled trials are still needed to further verify the efficacy of the two drugs and evaluate combination or sequential treatment regimens.

## Conclusion

Our findings suggest a stratified approach. Praziquantel remains the established first-line therapy. However, for suspected, stool-negative cases, Albendazole may serve as a potential empirical option, showing similar lesion improvement in this specific cohort. These findings are hypothesis-generating and warrant validation in prospective trials.

## Data Availability

The data analyzed in this study is subject to the following licenses/restrictions: Data are not publicly available due to patient privacy and institutional ethics restrictions. They can be obtained from the corresponding authors upon reasonable request. Requests to access these datasets should be directed to Jing Zhang, zhangj375@mail.sysu.edu.cn.
